# Long-Term Mortality Following Transvenous Lead Extraction: Data from EXTRACT Registry

**DOI:** 10.3390/jcm15176639

**Published:** 2026-08-28

**Authors:** Sylwia Gładysz-Wańha, Michał Joniec, Wojciech Wańha, Eugeniusz Piłat, Anna Drzewiecka, Rafał Gardas, Jolanta Biernat, Andrzej Węglarzy, Danuta Łoboda, Joanna Stachańczyk, Krzysztof Stanisław Gołba

**Affiliations:** 1 Department of Electrocardiology and Heart Failure, Medical University of Silesia, 40-635 Katowice, Polandkgolba@sum.edu.pl (K.S.G.); 2 Department of Electrocardiology, Upper Silesian Medical Center, Medical University of Silesia, 40-635 Katowice, Poland; 3 Department of Cardiology and Structural Heart Diseases, Medical University of Silesia, 40-635 Katowice, Poland; wojciech.wanha@gmail.com; 4 Department of Anaesthesiology and Intensive Care with Cardiac Supervision, Upper Silesian Medical Center, Medical University of Silesia, 40-635 Katowice, Poland; 5 Department of Cardiac Rehabilitation, Murcki Hospital, 40-749 Katowice, Poland

**Keywords:** transvenous lead extraction: cardiac implantable electronic device, mortality, infection, CHA_2_DS_2_-VA score, kidney function

## Abstract

**Background/Objective**: The EXTRACT registry cohort of 504 patients undergoing transvenous lead extraction (TLE) has been previously reported in terms of periprocedural safety and short-term outcomes. Data on long-term mortality and its predictors in this population remain limited. The study aimed to identify risk factors for overall mortality at three years following TLE. **Methods**: This is a secondary, long-term follow-up analysis of the previously published EXTRACT registry cohort of 504 consecutive patients who underwent TLE between 2016 and 2022. We conducted a univariate analysis to predict mortality risk following TLE, followed by Cox regression with cross-validation to identify independent predictors of mortality over a 3-year follow-up period; Brier score assessed prediction accuracy for mortality; and decision curve analysis evaluated the clinical usefulness of the model. **Results**: Mortality data were completed for 487 patients at three-year follow-up. Long-term outcomes of TLE showed that patients with CIED infection (*p* = 0.0091), higher NYHA class (*p* < 0.0001), arterial hypertension (*p* = 0.0068), previous stroke (*p* = 0.0092), upgraded to condition pacing post-TLE (*p* = 0.0092), and higher CHA_2_DS_2_-VA score (*p* = 0.0026) had markedly higher mortality at three-year follow-up. Increased mortality was independently associated with estimated glomerular filtration rate (eGFR) and the CHA_2_DS_2_-VA score. Risk increased incrementally with each 1 mL/min/1.73 m^2^ decrease in eGFR (hazard ratio, 0.99; 95% CI, 0.98–0.99), and each 1-point increase in CHA_2_DS_2_-VA conferred a 12% higher mortality rate (hazard ratio, 1.12; 95% CI, 1.02–1.23). **Conclusions**: In this prospective registry, 3-year mortality after TLE was driven predominantly by baseline clinical risk rather than procedural factors. Renal dysfunction and a higher CHA_2_DS_2_-VA score independently predicted death, supporting the incorporation of kidney function and global vascular risk into pre-extraction assessment and post-extraction management.

## 1. Introduction

Transvenous lead extraction (TLE) is an increasingly established procedure for the management of malfunctioning or device-related infections of cardiac implantable electronic devices (CIEDs). The periprocedural safety and efficacy of TLE in the EXTRACT Registry, including 30-day outcomes in infected and non-infected patients, have been published previously [1]. Importantly, TLE treatment remains challenging. It is due to a growing population, which keeps patients with implanted devices longer, leading to more cases of lead dysfunction or device infections [2]. Despite improvements in care, device-related infections (e.g., endocarditis, pocket abscess, sepsis, device erosion) remain among the most common causes of TLE [3]. TLE technologies have advanced in the last decade. Data from controlled clinical trials suggest that TLE constitutes an attractive therapeutic strategy in this setting [4,5]. Therefore, several questions regarding the long-term clinical efficacy and safety of TLE remain inadequately addressed. Moreover, the predictors of clinical outcomes are still unclear. Large real-world studies are crucial for establishing optimal TLE therapy and for developing objective, evidence-based criteria for determining death during follow-up. The present study is a secondary, long-term analysis and aimed to identify risk factors for overall mortality at three years following TLE.

## 2. Methods and Definitions

The EXTRACT Registry [Effectiveness, Complications, and Mortality of TLE in Patients] is a large observational study conducted in a high-volume TLE center. This data included 504 consecutive patients with CIEDs treated with TLE between January 2016 and May 2022 at the Department of Electrocardiology and Heart Failure at Medical University of Silesia (Katowice, Poland). The present analysis is a secondary analysis of this cohort focused on three-year all-cause mortality. Mortality data of 487 patients were obtained from the central database of the Polish National Health Fund, and seventeen patients were lost to follow-up (no record linkage possible in the National Health Fund database). STROBE flow diagram is provided in Supplemental Figure S1, summarizing patient selection, exclusion criteria, and follow-up status. The institutional review board approved the study protocol. The patients’ data were protected in accordance with Polish law, the GDPR (General Data Protection Regulation), and hospital Standard Operating Procedures. The study was conducted in accordance with the Declaration of Helsinki and was registered at www.ClinicalTrials.gov (NCT05775783).

The primary outcome was all-cause mortality at 3 years following TLE. Time zero was defined as the date of the index TLE procedure. Patients were followed from time zero until death or 3 years (1095 days) after TLE, whichever occurred first. Patients alive at 3 years were censored at that time point. Cumulative numbers of deaths were tabulated at 1 year, 2 years, and 3 years after TLE for the overall cohort. These event numbers are reported in Supplemental Table S1. All definitions were adapted from the 2026 HRS Expert Consensus Statement and are shown in Table 1.

## 3. Procedure Description

All extraction procedures were performed in the hybrid catheterization laboratory operating room. The team included certified electrophysiologists, cardio-anesthesiologists, cardiologists with experience in perioperative transesophageal echocardiography, nurses, and a radiology technologist. A cardiac surgery team was available onsite. The routine approach for the TLE procedure was the single traction method. If necessary, then the following methods were used: nonlocking and locking stylets (Liberator^®^ Beacon^®^ Tip Locking Stylet, Cook Medical, Leechburg, PA, USA); mechanical dilators (Byrd, Cook Vascular Inc., Leechburg, PA, USA); and powered rotational mechanical extraction sheaths (Evolution Mechanical Dilator Sheath or Evolution RL Mechanical Dilator Sheath, Cook Medical). A side loop (Needle’s EyeSnare, Cook Medical) was used for the femoral approach when the subclavian-only approach was insufficient. Laser techniques were not used.

### Statistical Analysis

Clinical characteristics were reported as counts and percentages of the total number of patients for categorical variables, and as mean ± SD or median (IQR) for continuous variables, depending on the distribution. The following preprocedural or periprocedural candidate variables were used as modified from Heart Rhythm Society expert consensus on CIED lead management [6]: body mass index (BMI), estimated glomerular filtration rate (eGFR), left ventricular ejection fraction (LVEF), New York Heart Association functional class (NYHA), infection as an indication for TLE, arterial hypertension (HA), atrial fibrillation (AF), coronary artery disease (CAD), diabetes mellitus (DM), previous stroke, pulmonary disease, CHA_2_DS_2_-VA score, clinical success, complete procedural success, upgrade to high-voltage therapy or conduction system pacing, adjusted to age and sex. Two missing eGFR values were imputed using the MICE method %. No patient in the analyzed cohort had a CHA_2_DS_2_-VA score of 0; observed scores ranged from 1 to 8.

We used the Kaplan–Meier estimator with the log-rank test as the univariate analysis to assess the hazard of mortality over 3 years after TLE for each binary or ordinal candidate variable. Continuous variables and variables significant in the Kaplan–Meier log-rank tests or showing a trend towards significance (*p* values between 0.05 and 0.1) were entered as candidate variables for the stepwise Cox proportional hazards model. It was used to identify independent predictors of mortality during the 3-year follow-up, and hazard ratios (HRs) and 95% CIs were calculated. Internal validation was performed using bootstrap resampling and k-fold cross-validation. The Brier score with confidence intervals was used to assess the accuracy of predicted mortality risk. Decision curve analysis with confidence intervals was performed to evaluate the model’s clinical usefulness. All reported associations are presented as hazard ratios (95% confidence intervals—95% CIs). The hazard ratio for continuous variables shows the relative change in endpoint risk per unit increase. To evaluate the ability of the independent predictors to discriminate 3-year mortality, we constructed receiver operating characteristic (ROC) curves using 3-year vital status as a binary endpoint. From this ROC curve, optimal cut-off values for independent predictors were defined, and the area under the ROC curve (AUC) with 95% CIs was calculated.

The criterion for statistical significance was set at *p* < 0.05. Stata Version 17 (StataCorp LLC, 2020, College Station, TX, USA), MedCalc^®^ Statistical Software Version 23.5.5 (MedCalc Software Ltd., Ostend, Belgium, 2026) and Python 3.14.2 with Pandas 2.3.3 were applied.

## 4. Results

### 4.1. Baseline and Procedural Characteristics

A total of 487 patients who underwent TLE with complete follow-up data were included in the pooled analysis, of whom 142 (29.2%) had CIED infection. The cohort was 34.5% female, and the median age of the total patients was 69 years (IQR 61–75 years). The median LVEF was 47% (IQR 31–58%), and the mean eGFR was 68.26 ± 19.67 mL/min/1.73 m^2^. Among the 487 patients, the most common was NYHA class II (30.8%), and the least common was NYHA class IV (1.2%). Comorbidities were as follows: HA 70.2%; AF 43.1%; CAD 52.6%; DM 29.2%; previous stroke 6.4%, and pulmonary disease 9%. Investigated CHA2DS2-VA scores were as follows, for scores of 1 through 8, respectively: 9.9%, 13.8%, 20.5%, 24.2%, 13.1%, 5.1%, 2.1%, 0.8%. Clinical success was achieved in 95.7% of patients, with a 92.2% complete procedural success rate. After the TLE, 14.6% of patients underwent an upgrade to high-voltage therapy, and 9% underwent an upgrade to conduction system pacing. Baseline and procedural characteristics are presented in Table 2.

Procedurally, the longest lead dwell time was 7 (IQR 4–11) years. Both single-traction and mechanical sheath systems were used in 67.6% of patients, whereas hand-powered rotational mechanical extraction sheath systems were used in 12.9%, side loop in 4.3%, and locking style in 67.6%, respectively. Procedural data are listed in Supplemental Table S2. Pacemakers were extracted in 59.3% of cases, and the most frequently removed lead was the right ventricular lead (99.6%). The type of targeted devices and leads is presented in Supplemental Table S3.

### 4.2. Long-Term Mortality

**Figure 1:**
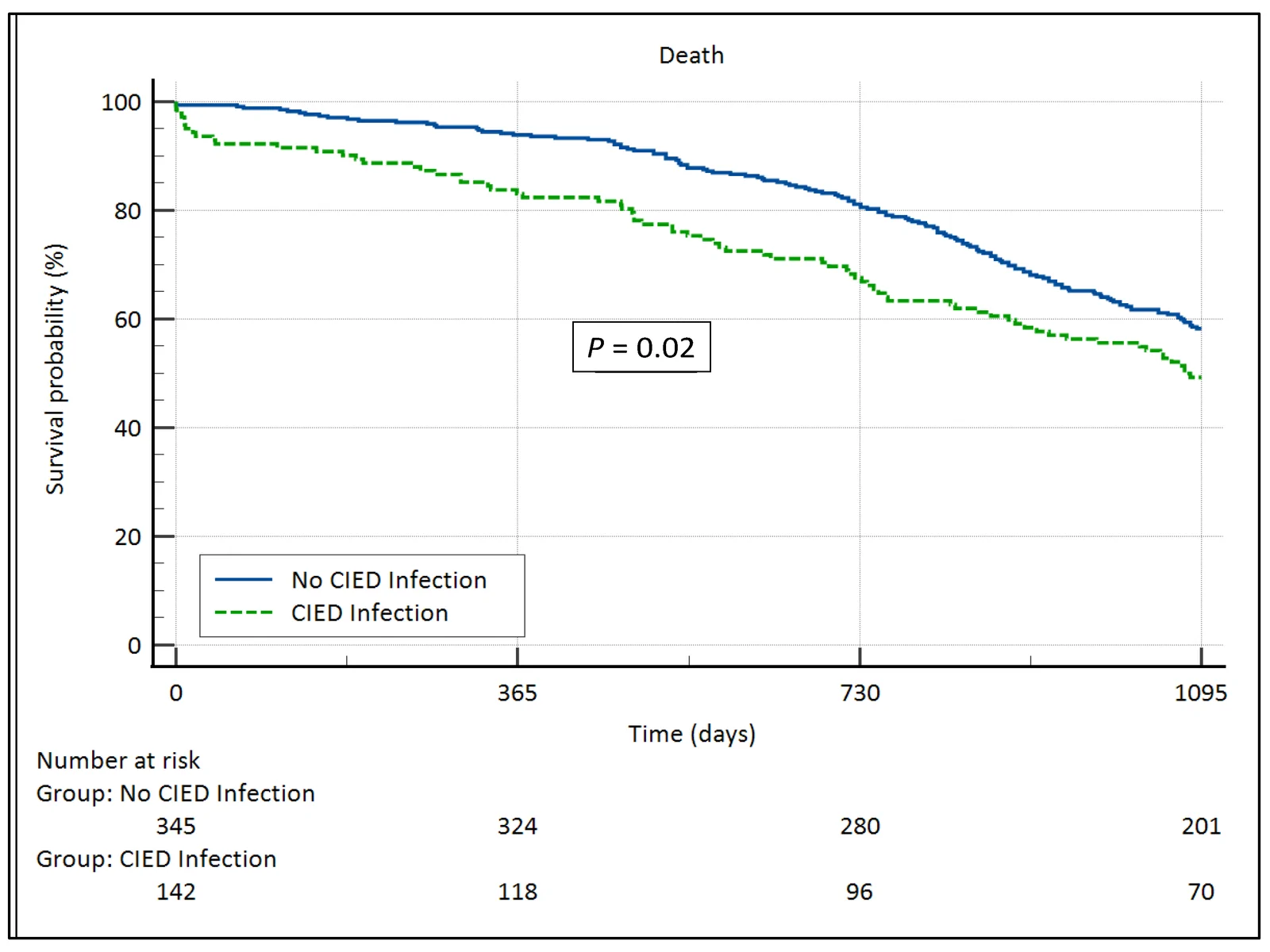
The Kaplan–Meier estimator curves for survival from all-cause death at 3-year follow-up in patients with or without cardiac implantable device (CIED) infection after transvenous lead extraction.

**Figure 2:**
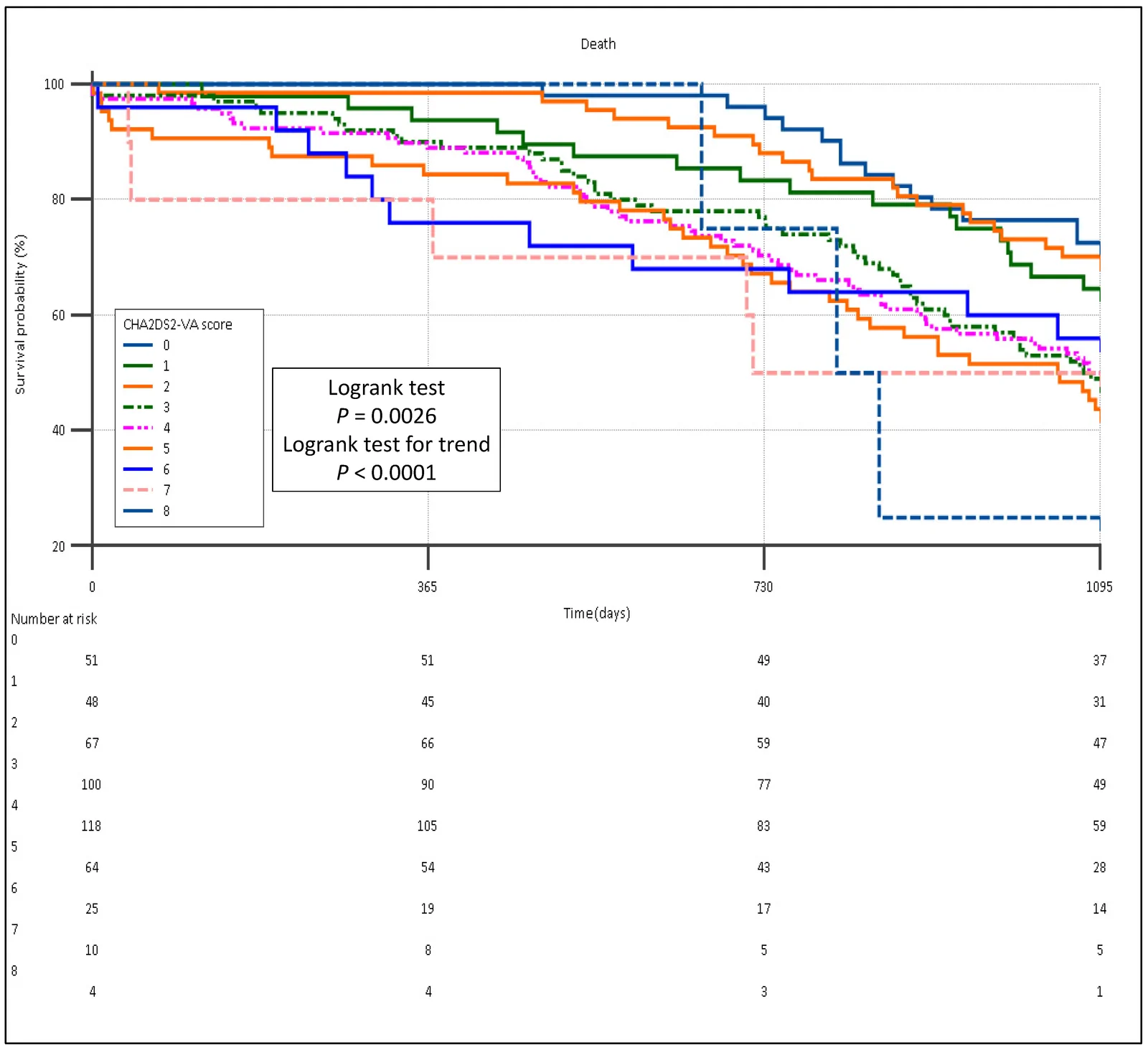
The Kaplan–Meier estimator curves of overall survival stratified by CHA_2_DS_2_-VA score after transvenous lead extraction.

**Figure 3:**
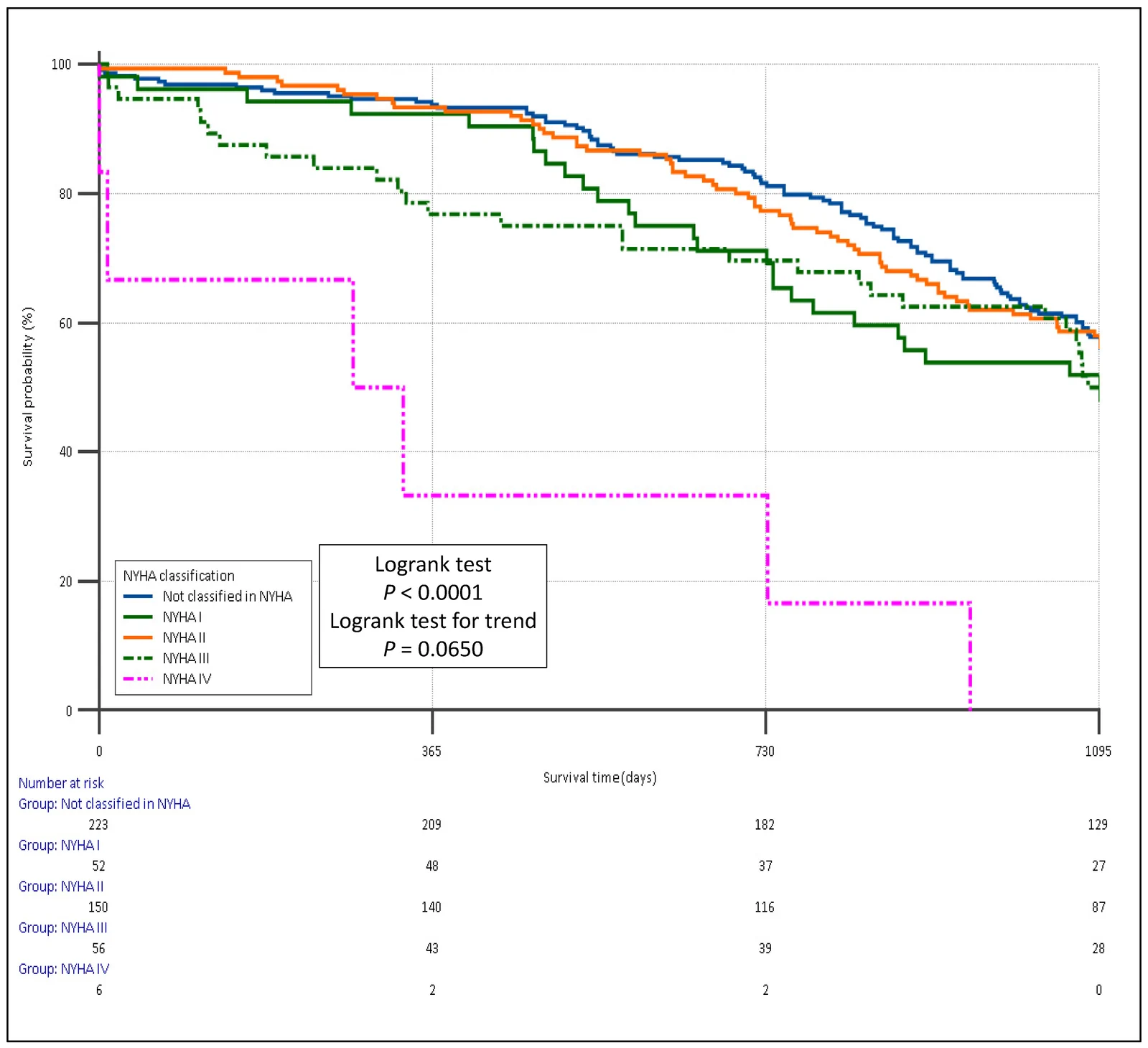
The Kaplan–Meier estimator curves of overall survival stratified by New York Heart Association (NYHA) class after transvenous lead extraction.

**Figure 4:**
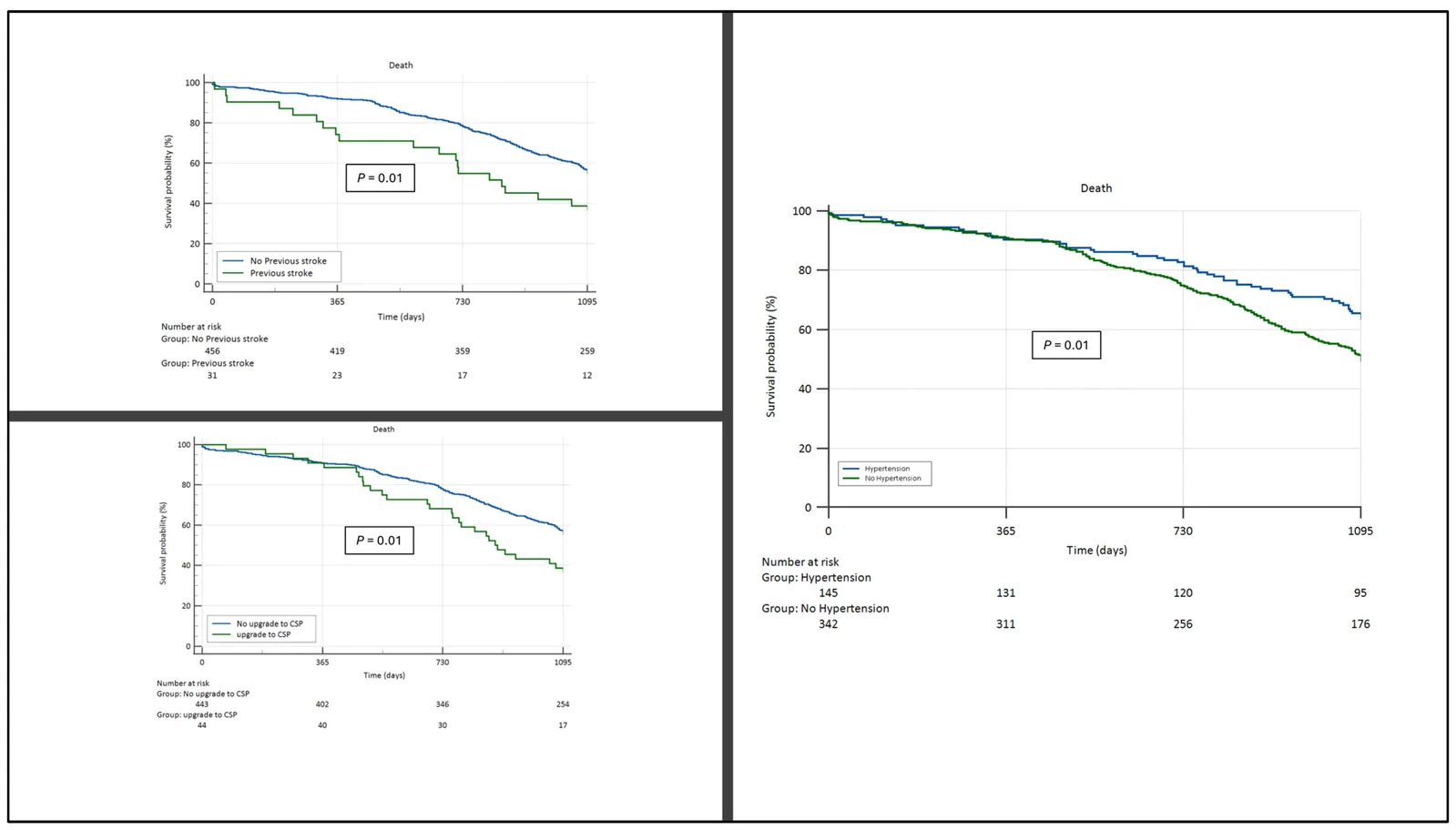
The Kaplan–Meier curves of overall survival stratified by arterial hypertension, previous stroke, and upgrade to conduction system pacing (CSP) after transvenous lead extraction.

**Figure 5:**
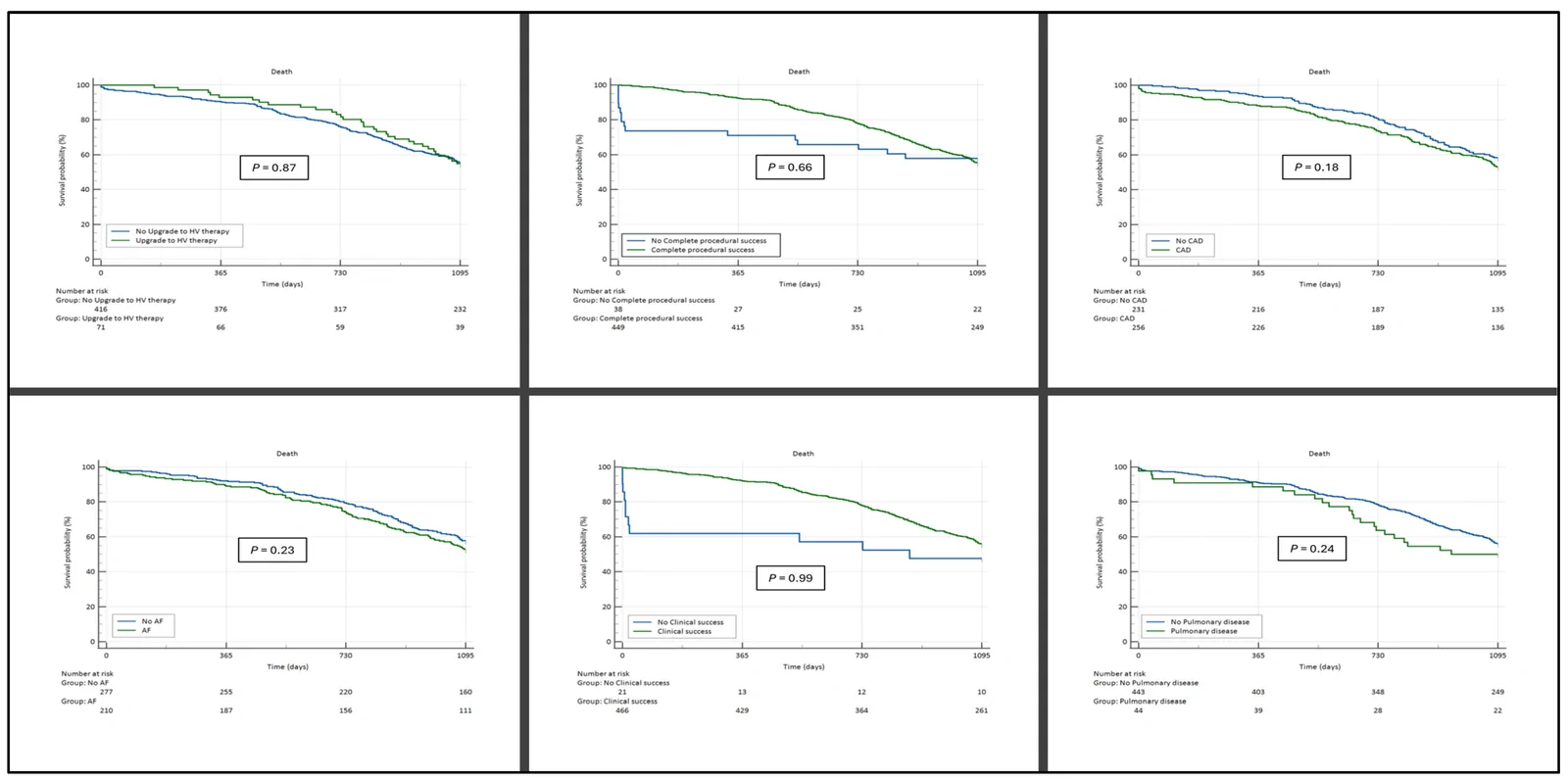
The Kaplan–Meier curves of overall survival stratified by upgrade to high-voltage therapy (HV) after TLE, atrial fibrillation (AF), complete procedural success, clinical success, coronary artery disease (CAD) and pulmonary disease.

Overall, 216 of 487 patients (44.35%) died within 3 years (1095 days). Three-year mortality was 50.7% among patients with CIED infection and 41.74% among those without CIED infection (*p* = 0.02). CIED infection was associated with higher mortality than a non-infectious indication (HR 1.43, 95% CI 1.06–1.95). Kaplan–Meier curves for all-cause death are presented in Figure 1. Table 3 presents the univariable analysis of predicted mortality after TLE. Moreover, our results showed that a higher CHA_2_DS_2_-VA score was associated with lower survival over 3 years (see Figure 2). The study also found significant differences in survival rates based on NYHA class. However, the non-significant trend test may have been influenced by small numbers in the NYHA IV group and by the presence of the “not classified” group (Figure 3). Additional Kaplan–Meier curves illustrating significant differences in three-year survival for patients with arterial hypertension, a history of stroke, and the presence of a CSP (His bundle branch/Left bundle branch pacing) lead are shown in Figure 4. Variables included in the model and subjected to univariate analysis, which did not show significant differences in patient mortality, are presented in Figure 5.

### 4.3. Predictors of Death

**Figure 6:**
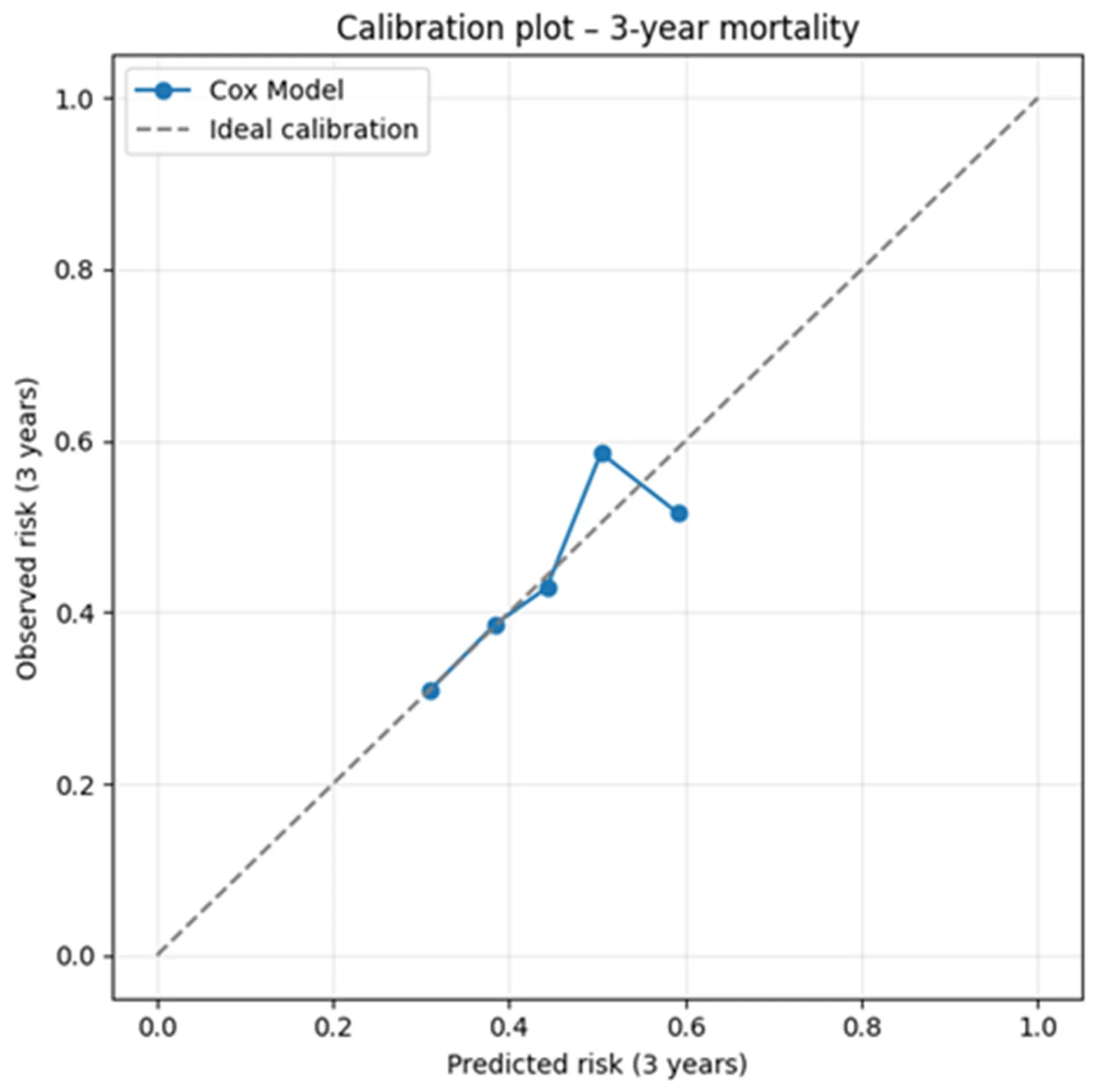
Decision Curve Analysis for Model Performance.

**Figure 7:**
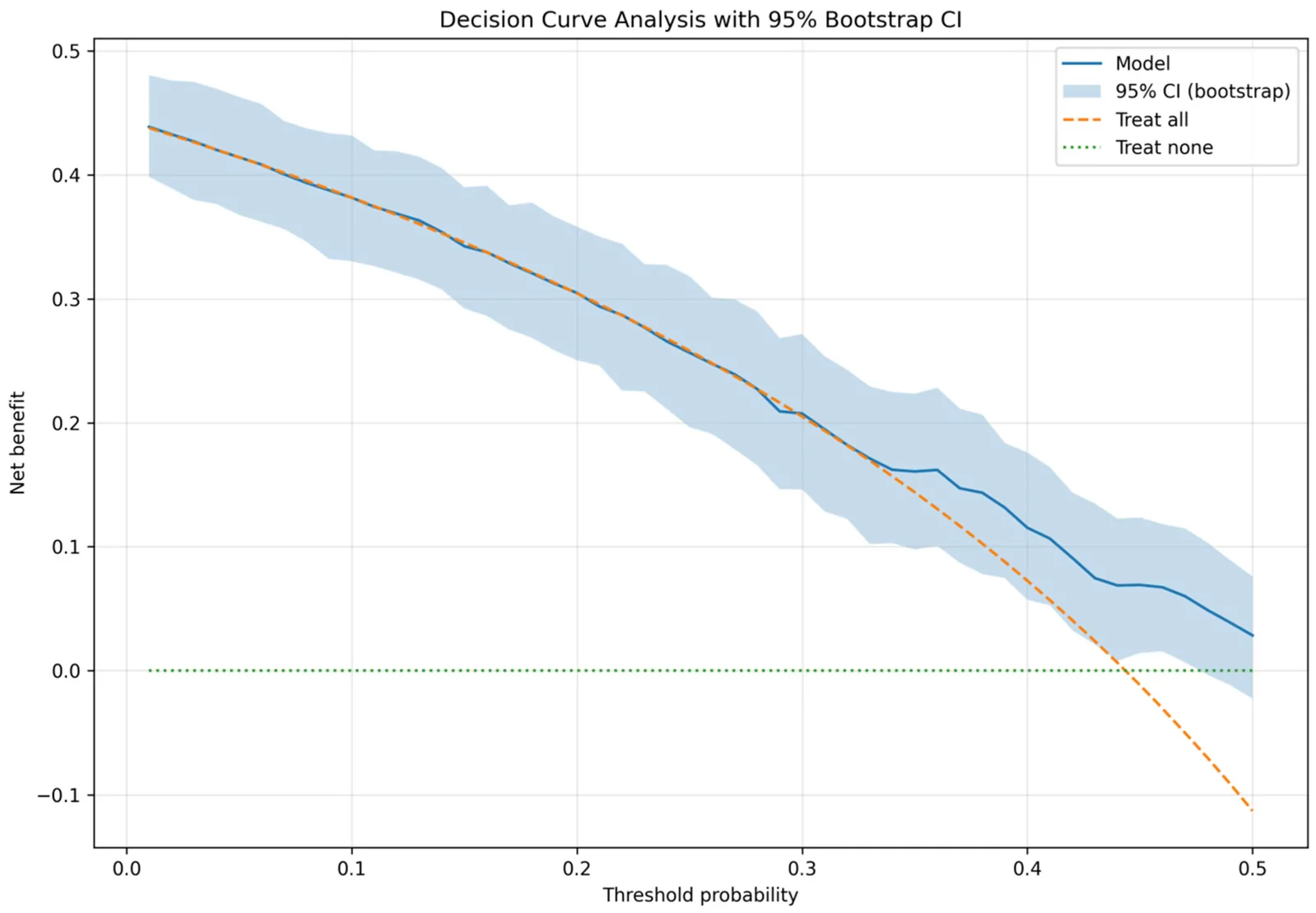
Decision Curve Analysis (DCA) for 3-year mortality predicted by the Cox model.

A stepwise Cox proportional hazards model identified two independent predictors of death in patients after TLE: eGFR and CHA_2_DS_2_-VA score. Variables not included in the model were HA, DM, LVEF, NYHA class, previous stroke, infection as a cause for TLE, BMI, clinical success, and age or female sex. The results were as follows: each 1 mL/min/1.73 m^2^ increase in eGFR is associated with about a 1% relative reduction in mortality (HR, 0.99; 95% CI, 0.98–0.99). Furthermore, each point increase in the CHA_2_DS_2_-VA score is associated with a 12% increase in mortality (HR, 1.12; 95% CI, 1.02–1.23). After dichotomizing the cohort, the cut-off values for eGFR and the CHA_2_DS_2_-VA score were 52 mL/min/1.73 m^2^ or lower and 5 or higher, respectively. Results are presented in Table 4 and Table 5. Furthermore, multivariate Cox regression of our data demonstrated a predictive model of 3-year mortality risk with an AUC of 0.608 (*p* = 0.0171) as confirmed by a Calibration Plot with Bootstrap validation (Table 6, Figure 6 and Figure 7). Predicted risk ranged from 0.593 to 0.611. The model’s accuracy in predicting actual mortality risk was confirmed by the Brier score: 0.0816 at 1 year, 0.1675 at 2 years, and 0.2379 at 3 years.

## 5. Discussion

The current analysis of the EXTRACT Registry examined long-term outcomes and factors associated with mortality after TLE in CIED-infection and non-CIED-infection patients in a real-world population. The main findings are as follows: (1) At the three-year follow up, mortality rate was 50.7% of patients with CIED infections, compared with 41.74% in patients with non-CIED infections. (2) Patients undergoing TLE with infection, higher NYHA class, hypertension, and previous stroke had a high mortality rate. (3) The eGFR and the CHA_2_DS_2_-VA score were found to be independent predictors of all-cause mortality in 3 years following TLE. Our results corroborate previous observational studies. While TLE can be performed safely in specialized centers, the long-term prognosis is heavily influenced by baseline patient comorbidities and indication, particularly infection [7,8]. Recent studies have also explored the feasibility and safety of same-day discharge in selected patients undergoing transvenous lead extraction. A 2023 multicenter analysis demonstrated that, in carefully screened low-risk patients, same-day discharge after TLE was associated with low complication rates and no increase in 30-day adverse events compared with standard in-hospital observation. These findings support the notion that TLE can be performed safely in experienced centers with appropriate patient selection and post-procedural pathways [9]. Our results, showing high procedural and clinical success rates with low major complication rates, are consistent with this evolving paradigm. The present three-year follow-up results on mortality in patients with CIED and non-CIED infections provide valuable data showing that TLE for cardiac device infection is associated with substantially higher mortality, and that both infection-related and patient-related factors can predict risk. Our data demonstrated that the incidence of infection further amplifies long-term death risk, as noted in other large studies [10,11]. Previous studies, including meta-analyses, have shown that patients with renal insufficiency experience an increase in all-cause mortality following TLE compared to patients without chronic kidney disease (CKD), with a hazard ratio of 2.14 (95% confidence interval 1.65–2.77) [12]. The current study also supports these findings. It should be noted that, traditionally, when assessing mortality risk in the general population and in patients with cardiovascular disease, eGFR becomes a clear prognostic risk factor only once it falls below about 60 mL/min/1.73 m^2^ [13]. However, recently it has been noted that, when assessing mortality risk in the context of the eGFR cut-off value, the risk may begin to increase at eGFR values just below 90–100 mL/min/1.73 m^2^ in younger individuals. In contrast, in elderly individuals, the prognostic threshold is closer to 45, arguing against a single universal cut-off [14]. Patients in our registry have a median age of 69 years. Their calculated eGFR cut-off value was 52 mL/min/1.73 m^2^, which is slightly below 60 mL/min/1.73 m^2^. Furthermore, patients with CKD undergoing TLE often have a higher burden of comorbidities such as heart failure, diabetes, and vascular disease, which independently worsen their prognosis [15]. Additionally, CKD is strongly linked to a higher risk of device infections and sepsis, which may necessitate extraction [16]. Global vascular risk, assessed by the CHA_2_DS_2_-VA score, was identified as an independent predictor of mortality in our cohort. Our findings are consistent with a recent large registry of 3822 patients undergoing non-laser TLE, which demonstrated a strong, graded association between CHA_2_DS_2_-VASc and CHA_2_DS_2_-VA scores and both short- and long-term mortality. In that study, each 1-point increase in the CHA_2_DS_2_-VA score was associated with an approximately 18% higher risk of death, which is numerically similar to the 12% increase observed in our cohort (HR 1.12; 95% CI 1.02–1.23). The novelty of our work lies in the detailed 3-year landmark mortality analysis, time-to-event modeling, and development of an internally validated exploratory prognostic model incorporating eGFR and CHA_2_DS_2_-VA in a well-characterized, single-center cohort with follow-up. While the larger registry provides robust evidence across multiple high-volume centers, our analysis offers complementary granular data on procedural characteristics, lead dwell time, and post-TLE management, which may inform risk stratification in similar settings [17]. To our knowledge, there is no dedicated, validated “CHA2DS2-VA-TLE” score or online mortality calculator yet. All components of CHA2DS2-VA (age, chronic heart failure, hypertension, stroke/TIA, vascular disease, diabetes) are responsible for systemic frailty and cardiovascular comorbidity that worsen the prognosis for patients undergoing major procedures such as TLE. The CHA2DS2-VASc score is a widely studied topic in publications on multimorbidity in patients. It is a significant predictor of mortality and adverse cardiovascular outcomes across various cardiovascular diseases, including ischemic stroke, heart failure, chronic kidney disease, chronic obstructive pulmonary disease, and acute coronary syndrome [18,19,20]. A study by Melgaard et al. evaluating the CHA2DS2-VASc score’s ability to predict ischemic stroke, thromboembolism (TE), and death in heart failure (HF) patients with and without atrial fibrillation (AF) demonstrated that higher CHA2DS2-VASc scores were associated with a higher risk of individual endpoints [21]. Furthermore, a study of over 165,000 patients showed that the CHA2DS2-VASc score is associated with mortality, regardless of AF presence or anticoagulation status [22]. The CHA2DS2-VASc score is also very useful for risk stratification in patients undergoing percutaneous coronary intervention (PCI) and carotid artery stenting (CAS) [23,24]. Therefore, the use of cardiac risk scores (e.g., heart failure status, CKD, CHA2DS2-VASc) can identify patients at highest risk of post-TLE mortality who warrant careful monitoring and follow-up. It is worth noting that Mehta et al. confirmed in their study that long-term mortality after TLE is more dependent on the severity of heart disease and comorbidities than on lead extraction alone [25]. A recently published study with data from the EXTRACT registry examines complications following TLE by age, with only modest age-related differences in periprocedural and short-term outcomes. Age is a component of the CHA2DS2-VA score. Therefore, further studies based on age-stratified patient cohorts, calculating their CHA2DS2-VA scores, could contribute to a more accurate estimate of the risk of the lead extraction procedure and thus to a better assessment of the balance of benefits and risks [26]. Further research on the CHA_2_DS_2_-VA score as a predictor of mortality in patients after TLE is highly valuable, as it can provide complementary data from real-life settings. Moreover, alternative studies may refine their predictive accuracy by incorporating additional clinical parameters. It should be noted that CHA2DS2-VASc complements, rather than replaces, TLE-specific predictors of death, such as systemic infection, CKD, advanced HF, or hemodynamic instability, which often have a strong effect. Notwithstanding this compelling evidence, CHA2DS2-VA provides substantial prognostic information but should be considered alongside infection status and organ failure when assessing long-term mortality after TLE. In summary, although the model’s ability to differentiate is moderate, it can be described as exploratory and hypothesis-generating, and the analysis strengthens the evidence base for considering kidney function and overall vascular risk in long-term prognostic assessment after TLE.

## 6. Study Limitations

We confronted non-infection CIED patients with CIED infection patients, and the groups differed in baseline characteristics. In summary, although the model’s ability to differentiate is moderate, it can be described as exploratory and hypothesis-generating, and the analysis strengthens the evidence base for considering kidney function and overall vascular risk in long-term prognostic assessment after TLE. All procedures in our study were performed solely with mechanical tools. The group needing CSP upgrade was small; patients formed a clinically more complex subgroup—they were older, had a high CHA_2_DS_2_-VA score, poor kidney function, and frequent infection indications for TLE. This finding is exploratory and likely reflects patient selection and other confounding factors, so it should not be interpreted as proof that CSP increases mortality after TLE.

Unfortunately, we present data from a single-center, but high-volume TLE registry.

## 7. Conclusions

In this prospective registry of a real-life population, the 3-year mortality rate after transvenous lead extraction (TLE) was primarily influenced by baseline clinical risk factors rather than procedural aspects. Specifically, renal dysfunction and a higher CHA_2_DS_2_-VA score were found to be independent predictors of mortality. It emphasizes the importance of considering kidney function and overall vascular risk in both the pre-extraction assessment and post-extraction management. Understanding these predictive factors can help identify high-risk patients, thereby improving the effectiveness of the procedure and reducing complications associated with TLE.

## Figures and Tables

**Table 1:** Transvenous lead extraction: definitions.

Transvenous Lead Extraction	A Procedure in Which at Least 1 Lead Required Equipment and at Least 1 Lead Was Implanted for >1 Year
Procedural success	removal of all targeted leads and all lead material from the vascular space, without any permanently disabling complications or procedure-related death
Clinical success	removal of all targeted leads and lead material from the vascular space, or retention of a small lead fragment measuring less than 4 cm that does not negatively affect the intended clinical outcome of the procedure
Failure	neither procedural nor clinical success is achieved, or the occurrence of a permanently disabling complication or procedure-related death
Major complications	A complication that results in death, a life-threatening event, a permanently disabling condition, or requires major intervention, including emergency surgery, vascular repair, chest-tube drainage, or prolonged hospitalization
Minor complications	A procedure-related complication that does not meet the criteria for a major complication and does not result in permanent disability or death

**Table 2:** Baseline and procedural characteristics of 487 patients.

Age (years), median (IQR)	69 (61–75)
Female, *n* (%)	168 (34.5)
Body mass index (kg/m^2^), median (IQR)	27.61 (24.64–31.64)
eGFR (ml/min/1.73 m^2^), mean (SD)	68.26 ± 19.67
LVEF (%), median (IQR)	47 (31–58)
NYHA functional class I, *n* (%)	52 (10.7)
NYHA functional class II, *n* (%)	150 (30.8)
NYHA functional class III, *n* (%)	56 (11.5)
NYHA functional class IV, *n* (%)	6 (1.2)
NYHA class not determined	223 (45.8)
Infection as an indication for TLE, *n* (%)	142 (29.2)
Arterial hypertension *n* (%)	342 (70.2)
Atrial fibrillation (%)	210 (43.1)
Coronary artery disease, *n* (%)	256 (52.6)
Diabetes mellitus, *n* (%)	142 (29.2)
Previous stroke, *n* (%)	31 (6.4)
Pulmonary disease, *n* (%)	44 (9.0)
CHA_2_DS_2_-VA score = 1, *n* (%)	48 (9.9)
CHA_2_DS_2_-VA score = 2, *n* (%)	67 (13.8)
CHA_2_DS_2_-VA score = 3, *n* (%)	100 (20.5)
CHA_2_DS_2_-VA score = 4, *n* (%)	118 (24.2)
CHA_2_DS_2_-VA score = 5, *n* (%)	64 (13.1)
CHA_2_DS_2_-VA score = 6, *n* (%)	25 (5.1)
CHA_2_DS_2_-VA score = 7, *n* (%)	10 (2.1)
CHA_2_DS_2_-VA score = 8, *n* (%)	4 (0.8)
Clinical success, *n* (%)	466 (95.7)
Complete procedural success, *n* (%)	449 (92.2)
Upgrade to HV therapy, *n* (%)	71 (14.6)
Upgrade to CSP, *n* (%)	44 (9.0)

eGFR—estimated glomerular filtration rate; LVEF—Left Ventricular Ejection Fraction; NYHA—New York Heart Association; TLE—Transvenous Lead Extraction; HV—high voltage; CSP—conduction system pacing.

**Table 3:** The Kaplan–Meier estimator with a log-rank test as the univariate analysis to assess mortality hazard over 3 years after TLE for a binary candidate variable.

Independent Predictors	*p*-Value	Hazard Ratio	95% CI
Arterial hypertension	0.0068	0.6726	0.5047–0.8962
Atrial fibrillation	0.2297	0.8472	0.6463–1.1105
Coronary artery disease	0.1784	0.8325	0.6374–1.0873
NYHA class	<0.0001		
NYHA class not determined		-	-
NYHA class I		0.7689	0.4817–1.2273
NYHA class II		0.9724	0.7155–1.3214
NYHA class III		0.7452	0.4742–1.1711
NYHA class IV		0.1393	0.0018–1.0845
Previous stroke	0.0092	0.4417	0.2387–0.8172
Pulmonary disease	0.2360	0.7426	0.4539–1.2148
Infection as an indication for TLE	0.0191	0.6959	0.5139–0.9423
Clinical success *	0.0991	1.9047	0.8857–4.0964
Procedural success *	0.6618	1.1269	0.6598–1.9248
CHA2DS2VA score	0.0026		
CHA2DS2VA score 1		0.7185	0.4118–1.2537
CHA2DS2VA score 2		0.8918	0.5368–1.4816
CHA2DS2VA score 3		0.4432	0.2733–0.7187
CHA2DS2VA score 4		0.4355	0.2716–0.6984
CHA2DS2VA score 5		0.3696	0.2145–0.6370
CHA2DS2VA score 6		0.4847	0.2351–0.9992
CHA2DS2VA score 7		0.3751	0.1242–1.330
CHA2DS2VA score 8		0.2593	0.0049–1.3583
Upgrade to HV	0.8708	1.013	0.7112–1.4957
Upgrade to CSP	0.0092	1.9407	1.1781–3.1968

NYHA—New York Heart Association; HV—high-voltage therapy; CSP—Conduction system pacing (His bundle pacing or left bundle branch pacing), *—according to 2017 HRS expert consensus statement on cardiovascular implantable electronic device lead management [6], repeated in the 2026 update HRS.

**Table 4:** The Cox proportional hazards model—independent predictors of mortality during the 3-year follow-up after TLE.

eGFR	0.99 (0.98–0.99)	0.04
CHA_2_DS_2_-VA score	1.12 (1.03–1.23)	0.01

Variables not included in the model: Arterial hypertension; Diabetes mellitus; LVEF; NYHA; Previous stroke; Infection as an indication to transvenous lead extraction; Age, Female sex; BMI; Clinical success; Upgrade to conduction system pacing after TLE.

**Table 5:** Cut-off values for independent predictors obtained from ROC curve analysis for fixed 3-year mortality, with comparisons (log-rank test) using secondary Kaplan–Meier estimations after dichotomizing the cohort at those cut-offs.

	Cut-Off Value	Sensitivity	Specificity	AUC (95% CI)	*p* for AUC	Log-Rank Test *p*
eGFR	≤52	28.14	81.32	0.591 (0.546–0.635)	0.0004	0.012
CHA_2_DS_2_-VA score	>4	25.46	82.29	0.598 (0.553–0.642)	0.0006	0.0035

eGFR—Glomerular Filtration Rate (ml/min/1.73 m^2^), AUC—Area Under the ROC curve.

**Table 6:** Net benefit of the prediction model at clinically relevant threshold probabilities (bootstrap 95% CI).

Threshold	NB Model (Mean)	95% CI	NB Treat-All	Gain vs. Treat-All	Conclusion
**0.30**	0.207	0.146–0.272	0.205	+0.0006	Start of model advantage
**0.35**	0.161	0.098–0.223	0.144	+0.015	Estimated net benefit compared with the treat-all strategy
**0.36**	0.162	0.101–0.228	0.131	+0.030	**CI entirely above treat-all**
**0.40**	0.115	0.057–0.176	0.073	+0.041	Estimated net benefit compared with the treat-all strategy
**0.45**	0.069	0.014–0.124	−0.012	+0.079	Treat-all becomes harmful
**0.50**	0.028	−0.023–0.076	−0.113	+0.140	Estimated net benefit compared with the treat-all strategy

## Data Availability

The original contributions presented in this study are included in the article/Supplementary Materials. Further inquiries can be directed to the corresponding author.

## Supplementary Materials

The following supporting information can be downloaded at: https://www.mdpi.com/article/10.3390/jcm15176639/s1.
