# Supplementary material for: Long-Term Mortality Following Transvenous Lead Extraction: Data from EXTRACT Registry
Source: J Clin Med. 2026 Aug 28;15(17):6639. doi: 10.3390/jcm15176639 (PMC13566713; doi:10.3390/jcm15176639)
Supplement: Supplementary file 1 [file jcm-15-06639-s001.zip › jcm-4502114-supplementary.pdf]

Supplemental Figure S1.

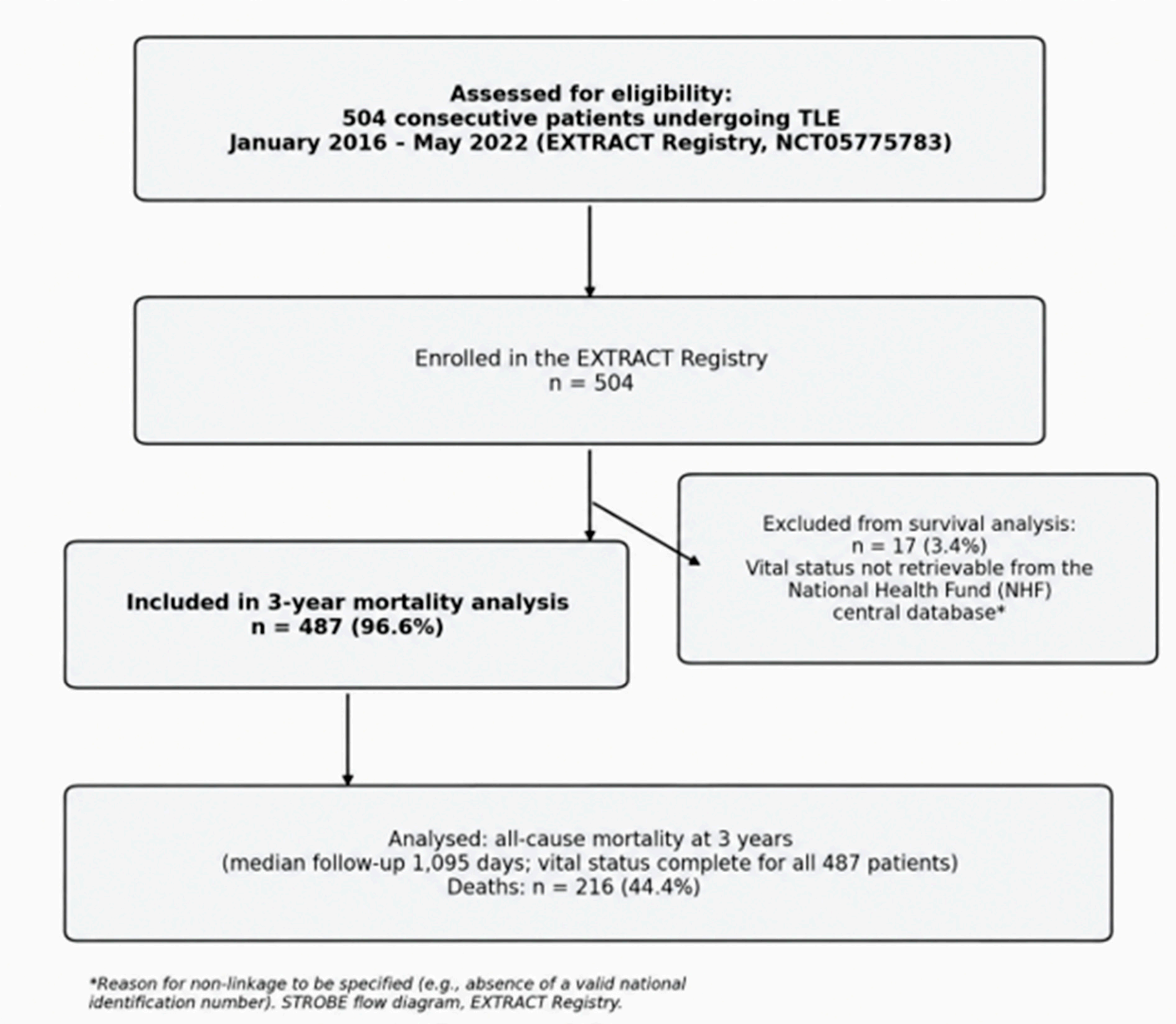

Supplemental Table S1.

Time after TLE procedure	Deaths, n (%)
1 year	45 (9.24%)
2 years	112 (23.0%)
3 years	216 (44.35%)

All-cause mortality during follow-up after transvenous lead extraction. All-cause mortality was assessed at 30 days and at 1, 2, and 3 years following the index transvenous lead extraction (TLE) procedure. Data are presented as n (%), with percentages calculated using the final analytical cohort of 487 patients as the denominator.

Supplemental Table S2. Procedural data of 487 patients

Lead dysfunction (including lead fracture or insulation failure resulting in abnormalities of impedance, sensing or capture), n (%)	282 (57.9)
Need to use a different stimulation type, n (%), upgrade to:	167 (34.3)
ICD, n (%)	36 (7.4)
CRT-D, n (%)	35 (7.2)
CRT-P, n (%)	64 (13.1)
CSP, n (%)	44 (9.0)
Longest lead dwell time (years), median (IQR)	7 (4-11)
Total amount of extracted lead, mean (SD)	1.96 ± 0.70
Single traction only, n (%)	158 (32.4)
Both single traction and mechanical sheath systems, n (%)	329 (67.6)
Hand-powered rotational mechanical extraction sheaths, n (%)	63 (12.9)
Side loop, n (%)	21 (4.3)
Locking stylet, n (%)	329 (67.6)
Simultaneous reimplantation on the extraction side (left subclavian approach), n (%)	323 (66.3)
Simultaneous reimplantation on the right side (right subclavian approach), n (%)	17 (3.5)
Vacuum-assisted wound closure systems, n (%)	4 (0.8)
Duration of the hospital stay (days), median (IQR)	6 (5-10)
ICD-implantable cardioverter-defibrillator; CRT-D–Cardiac resynchronization therapy defibrillator; CRT-P-cardiac resynchronization therapy with peacemaker function; CSP - Conduction system pacing (His bundle pacing or left bundle branch pacing).	

Supplemental Table S3. Preprocedural CIED system characteristics of 487 patients

PM, n (%)	289 (59.3)
PM RV leads, n (%)	79 (16.2)
PM RA leads, n (%)	248 (50.9)
ICD, n (%)	116 (23.8)
Defibrillator leads, n (%)	190 (39.0)
ICD RA leads, n (%)	59 (12.1)
CRT-P, n (%)	7 (1.4)
CRT-P RV leads, n (%)	7 (1.4)
CRT-P RA leads, n (%)	6 (1.2)
CRT-D, n (%)	74 (15.2)
CRT-D RV leads, n (%)	74 (15.2)
CRT-D RA leads, n (%)	57 (11.7)
Atrial lead, n (%)	371 (76.2)
Right ventricular lead, n (%)	485 (99.6)
Coronary sinus lead, n (%)	81 (16.6)
Non-functional leads, n (%)	43 (8.8)
CIED – cardiac implantable electronic device; PM - pacemaker; RV - right ventricle; RA - right atrial; ICD - implantable cardioverter-defibrillator; CRT-P - cardiac resynchronization therapy with a pacemaker; CRT-D - cardiac resynchronization therapy with a defibrillator.	
